# SCEM: A Structure-Preserving Privacy Framework for Sensor-Generated Time-Series Data

**DOI:** 10.3390/s26144477

**Published:** 2026-07-14

**Authors:** Shehui Jin, Qian Pu, Shankui Zheng, Haikuo Shen

**Affiliations:** 1 School of Mechanical, Electronic and Control Engineering, Beijing Jiaotong University, No. 3 Shangyuancun, Haidian District, Beijing 100044, China; 23115173@bjtu.edu.cn (S.J.);; 2 Henan Aerospace Rocket Co., Ltd., Jigu Road, Emergency Intelligent Manufacturing Industrial Park, No. 6 Factory Building, Qibin District, Hebi 458030, China

**Keywords:** sensor privacy, time series, IoT, feature inference, privacy–utility trade-off

## Abstract

Intelligent sensor systems and Internet of Things (IoT) platforms continuously generate high-value time-series data for downstream analytics and operational monitoring. However, statistical, spectral, and distributional characteristics embedded in these sequences may inadvertently expose sensitive behaviors, operating states, or temporal routines. To address this issue, we propose Structured Constrained Energy Mapping (SCEM), a general and extensible framework for the feature-selective protection of sensor-generated time-series data. Its generality arises from a unified instantiation interface that supports customizable protection mechanisms for heterogeneous sensitive functionals. SCEM follows a four-stage operator workflow, namely feature mapping, structured perturbation, feature recovery, and utility-oriented calibration, with a generalized privacy energy representation supporting the transition from mapping to perturbation. We demonstrate the practical applicability of the framework through five representative instantiations covering spectral amplitudes, mean, variance, quantiles, and high-order moments. Experiments on two sensor-related monitoring benchmarks and one non-sensor temporal benchmark show that SCEM achieves an unweighted mean attack success rate at the 10% reporting threshold (ASR@10%) of 4.69% over the 18 reported SCEM feature–dataset pairs under the evaluated direct feature inference attacks, indicating the reduced recoverability of predefined feature-level information in this protocol. Meanwhile, SCEM maintains competitive forecasting-oriented utility compared with representative protection baselines and the original-data utility reference, achieving a favorable privacy–utility trade-off. Overall, the results suggest that SCEM can serve as a practical model-free preprocessing layer for reducing feature-level leakage while preserving useful temporal structures for intelligent sensor data sharing.

## 1. Introduction

Sensor-generated time-series data are continuously produced by intelligent sensor networks, smart meters, industrial sensing systems, wearable and healthcare sensors, environmental monitoring sensors, and Internet of Things (IoT) sensing platforms [1,2]. These data provide a key foundation for edge and cloud sensor analytics, including forecasting, diagnosis, anomaly detection, monitoring, and operational decision-making. For instance, more than 1.2 billion smart meters had been deployed worldwide by 2023 [3], continuously collecting fine-grained energy consumption data, which may raise privacy concerns [4,5]. At the same time, modern deep learning models increasingly require high-quality temporal data, whose predictive value depends on temporal dependence, statistical regularity, and dynamic evolution [6,7]. This creates a fundamental tension between sensor-generated time-series data sharing and the protection of sensitive information.

The structural nature of sensor-captured time-series data makes this tension particularly difficult to manage [8,9]. Sensitive information is not necessarily stored in individual samples; it may be encoded in frequency-domain characteristics, statistical summaries, temporal correlations, local order statistics, or high-order moments [10,11]. Such feature-level leakage can have direct operational and personal consequences. In smart meter streams, periodic load patterns can reveal occupancy schedules and recurring household activities [12,13,14,15]. In industrial monitoring, shifts in statistical or spectral characteristics may expose production cycles, machine utilization patterns, or proprietary operating regimes [16]. In wearable and healthcare sensing, recurring spectral and distributional patterns may disclose health-related states or private behavioral routines [17]. These risks can remain present even when individual samples appear innocuous, because sensitive information may be encoded in aggregate, distributional, and periodic structures. Recent smart meter privacy studies also examine residential energy management and AMI analytics under privacy constraints [18,19]. Therefore, privacy protection for sensor-generated time-series data should not only reduce access to raw values but also control the inferability of sensitive features while preserving structures required for downstream sensor analytics. Feature-level inference therefore represents a practical data security risk in sensor data sharing, rather than merely an abstract statistical concern.

Existing privacy protection methods for sensor-generated time-series data have been developed from several perspectives, including anonymization, differential privacy, synthetic data generation, and frequency-domain perturbation [20,21,22,23]. Differential privacy has been widely studied and provides formal sensitivity-based protection mechanisms [24,25]; recent surveys emphasize that the central difficulty is to balance privacy protection with temporal utility [26]. Nevertheless, many existing approaches are either applied directly to raw samples, where raw-value perturbation may damage the temporal continuity and spectral regularity needed for sensor analytics, or are designed for a specific feature type, without an explicit operator-level interface for heterogeneous sensitive functionals [27]. A recent energy-aware time-series analysis further suggests that feature-space and energy-space representations can help to characterize temporal patterns [28], but intelligent sensor systems still require controllable privacy–utility trade-offs before data sharing or cloud analytics.

This paper proposes Structured Constrained Energy Mapping (SCEM), a general and extensible operator interface framework for feature-selective privacy protection in sensor-generated time-series data. The central idea is to represent each sensitive functional through a feature-specific instantiation tuple, perturb the corresponding representation in a structured manner, and recover a feasible protected sequence through feature-specific back-projection and utility-oriented calibration. The five evaluated cases are representative instantiations used to illustrate its extensibility and practical applicability, serving as a foundation for broader feature-specific extensions within the SCEM architecture. This design enables the targeted control of sensitive features while constraining the unnecessary distortion of utility-related statistical, spectral, and temporal structures. The main contributions of this paper are summarized as follows:

(1) We formulate feature inference privacy for sensor-generated time-series data sharing. Instead of focusing only on raw-sample perturbation, this formulation emphasizes sensitive statistical, spectral, and distributional features that may reveal user behaviors, operating states, or temporal routines in sensing scenarios.

(2) We propose Structured Constrained Energy Mapping (SCEM), a unified operator interface framework for feature-selective privacy protection. SCEM organizes heterogeneous sensitive functionals through a shared four-stage workflow, including feature mapping, structured perturbation, feature recovery, and utility-oriented calibration, supported by a generalized privacy energy representation between mapping and perturbation. This design enables targeted feature protection while constraining the unnecessary distortion of utility-related temporal structures.

(3) We instantiate SCEM for five representative privacy targets, including spectral amplitudes, mean, variance, quantiles, and high-order moments. Experiments on two sensor-related monitoring benchmarks and one non-sensor temporal benchmark show that SCEM reduces feature-level leakage while maintaining competitive forecasting-oriented utility, providing initial evidence of its applicability beyond physical sensor benchmarks under the evaluated settings.

## 2. Related Work

### 2.1. Random Perturbation and Differential Privacy

Random perturbation and differential privacy (DP) are fundamental techniques for data privacy protection [29]. Random perturbation protects data by injecting stochastic noise, whereas DP provides a formal mechanism for bounding the influence of individual records through sensitivity-calibrated randomization. For sensor time-series data, however, direct perturbation in the raw sample space may be structure-agnostic [8,21]. When temporal dependence, spectral regularity, and forecasting-oriented utility are important, noise that is not aligned with the semantics of the protected feature may unnecessarily alter non-sensitive structures and reduce downstream performance [9,30,31].

This limitation does not diminish the theoretical value of DP. Rather, it highlights a complementary problem setting in which the privacy target is a predefined statistical, spectral, or distributional functional of a released sequence. In this setting, a useful protection mechanism should first identify the representation in which the target functional is controllable and then modify the corresponding privacy-related component while explicitly constraining utility-related structures. Raw-space noise injection does not, by itself, provide such a functional-specific operator interface. Moreover, a statistic-level DP mechanism and a structure-preserving feature obfuscation mechanism address different privacy goals: the former provides individual-level indistinguishability under a specified neighboring relation, whereas the latter reduces the recoverability of selected functionals under a specified feature inference threat model. These two directions are therefore complementary rather than interchangeable.

### 2.2. Time-Series Anonymization and Feature Protection

Sensor time-series anonymization and feature protection methods move beyond global random perturbation by exploiting temporal segmentation, clustering, or transformed-domain representations [9,31]. The k-anonymity-based k-nTS method constructs equivalence groups through segmentation and clustering and can preserve selected temporal patterns during anonymization [32,33]. Frequency-domain anonymization and spectral privacy methods are effective when leakage is concentrated in periodic or harmonic components [20,30]. Quantile-oriented and attribute-oriented methods further show that privacy objectives can be expressed through specific statistical targets rather than only through raw-value distortion [34,35].

These approaches are effective in their intended protection spaces, but their internal mechanisms are usually tied to a particular feature family. Segmentation and clustering do not directly specify how a selected mean, variance, quantile, or spectral amplitude should move toward a privacy target. Spectral masking provides explicit control in the frequency domain, but it does not directly define how order statistics or high-order distributional descriptors should be protected. Likewise, a quantile-specific mechanism does not automatically extend to periodic patterns or dispersion-related characteristics. Therefore, the main unresolved issue is not the absence of effective feature-specific methods but the absence of a common construction rule that explains how heterogeneous functionals can be represented, perturbed, recovered, and calibrated under one extensible framework.

### 2.3. Structure-Aware and Learning-Based Privacy Preservation

Structure-aware perturbation methods seek to reduce utility loss by modifying sensitive information in transformed feature spaces instead of perturbing all raw samples indiscriminately [10,11,36]. In intelligent sensor systems, protection mechanisms should preserve structures required by downstream tasks such as forecasting, monitoring, anomaly detection, and diagnosis [9,21]. Studies on multivariate time-series representation, including transformer-based and convolutional sequence models, also show that downstream usefulness depends strongly on retaining task-relevant temporal structures [37,38].

Modern learning-based privacy approaches address this problem from complementary perspectives. Federated learning keeps model training local, which is valuable in medical and IoT applications [39]. Learned representations, privacy-oriented representation learning, and synthetic-data or generative approaches reduce the exposure of original sequences or retain selected distributional properties [37,40,41]. These methods provide strong modeling capacity but generally require training, architecture selection, task-specific optimization, or repeated parameter tuning, and their protection objectives are often expressed at the model, latent representation, or synthetic distribution level rather than as the direct control of an explicitly selected functional.

Beyond temporal data privacy, multimedia security research likewise shows that data protection mechanisms should be evaluated against explicit adversarial models rather than only through visual or statistical distortion. A recent cryptanalysis of a chaos- and S-box-based video cryptosystem identified structural weaknesses under chosen-plaintext, known-plaintext, and chosen-ciphertext attacks [42]. Although multimedia encryption and feature-selective time-series protection address different security goals, this broader evidence reinforces the importance of clearly specifying the attacker’s knowledge, protection scope, and structural assumptions when designing data protection mechanisms.

SCEM is positioned as a model-free preprocessing framework that is complementary to federated learning, synthetic data generation, and privacy-preserving representation learning. It provides a direct operator interface for feature-selective modification before time-series data are published or supplied to an external analytical model.

Existing structure-aware methods nevertheless leave three mechanism-level gaps. First, many methods are coupled to a specific model, task, or representation rather than offering a reusable interface for heterogeneous sensitive functionals. Second, the conditions under which perturbation, reconstruction, and calibration produce a valid protected sequence are often implicit. Third, the roles of privacy modification and utility restoration are not always separated, making it difficult to determine whether protection arises from the intended feature perturbation or from subsequent clipping and correction. These gaps motivate an operator-level formulation in which each stage has an explicit responsibility.

### 2.4. Energy- and Feature-Space Representations

In sensor time-series analysis, energy-related quantities are widely used for feature extraction, anomaly detection, and distributional characterization [43]. Sensor signals are commonly described through frequency-domain amplitudes, squared deviations, statistical moments, and other transformed representations. For power-load and smart-metering data, privacy-relevant behavior may appear as localized spectral components or characteristic distributions that differ from non-sensitive background structures [40]. These observations suggest that privacy protection can be more targeted when it is performed in a representation aligned with the sensitive functional.

Energy-based and feature-space representations have also been explored in privacy-related generative modeling and synthetic smart-grid data analysis [40,41]. However, the term “energy” is used differently across spectral, statistical, and distributional settings. Spectral energy may refer to amplitude or power, variance-related energy may refer to squared centered deviations, and distributional protection may rely on a discrepancy between a feature representation and a target state. Without an explicit operational definition, combining these meanings can make a general framework difficult to interpret and test.

To avoid this ambiguity, SCEM uses a generalized privacy energy concept that is formally defined in Section 3. In SCEM, privacy energy is not assumed to be a universal physical quantity. It is defined as a non-negative, weighted discrepancy between a functional-dependent representation and its privacy reference. Spectral power, centered dispersion energy, and squared feature deviation are treated as different instantiations of this common construction. This definition separates the invariant framework-level role of energy from the functional-specific representation used in each protection mechanism.

### 2.5. Summary and Motivation

The preceding review reveals four mechanism-level limitations that motivate SCEM. First, raw-space random perturbation and statistic-level DP can provide important privacy protection, but they do not directly specify where a heterogeneous target functional should be represented or how non-target structures should be constrained. This motivates a functional mapping stage that makes the selected sensitive characteristic explicit in an appropriate representation space. Second, anonymization and feature-specific methods are usually effective only in a predefined protection domain, which limits their transfer from spectral features to means, variances, quantiles, or high-order moments. This motivates a generalized privacy energy construction and a structured perturbation stage whose internal realization is allowed to depend on the protected functional.

Third, transformed-domain modification must be paired with a recovery step that converts the perturbed representation into a valid time series. Frequency-domain masking, dispersion redistribution, local quantile adjustment, and moment shaping require different reconstruction rules. This motivates a separate recovery or back-projection stage. Fourth, privacy-oriented modification may introduce value range violations or unintended changes to non-sensitive statistical, spectral, and temporal structures. This motivates an explicit utility-oriented calibration stage governed by application-dependent constraints.

Accordingly, SCEM provides a common but customizable mapping–perturbation–recovery–calibration interface for heterogeneous sensitive functionals. Its scope is feature inference privacy for released time-series data, complementing rather than replacing individual-level DP, federated learning, and model-based synthetic data generation.

## 3. The Proposed SCEM Framework

### 3.1. Threat Model and Problem Formulation

Let $X={\left[x_{1},x_{2},\ldots ,x_{T}\right]}^{⊤}\in X\subseteq R^{T}$ denote a time-series segment to be released. The segment may be collected by a smart meter, an environmental sensor, an industrial monitoring device, a wearable sensor, or another sensing platform. The objective is to construct a protected sequence $X^{†}\in X$ that reduces the recoverability of predefined sensitive information while retaining the structures required for downstream temporal analytics.

The sensitive information is represented by a measurable functional

(1) $$\phi :X\to Y_{\phi },$$

where $Y_{\phi }$ may be scalar-, vector-, or representation-valued. Examples include the mean or variance, a selected quantile, a vector of high-order moments, a set of spectral amplitudes, an autocorrelation profile, or a domain-specific operational indicator. When several characteristics are considered jointly, they can be written as a vector-valued functional

(2) $$\phi \left(X\right)={\left[\phi_{1}\left(X\right),\phi_{2}\left(X\right),\ldots ,\phi_{K}\left(X\right)\right]}^{⊤}.$$

The representative experiments in this work instantiate one functional at a time in order to isolate the behavior of each protection mechanism, whereas the framework itself permits joint functionals through Equation (2).

The adversary is assumed to observe the released sequence $X^{†}$, know the type of the protected functional, and construct an estimator $\hat{\phi }=A_{\phi }\left(X^{†}\right)$ using the available domain knowledge. For a functional-dependent discrepancy $d_{\phi }$ and tolerance $\tau_{\phi }>0$, an inference attempt is regarded as successful when

(3) $$d_{\phi }\left(\hat{\phi },\phi \left(X\right)\right)\leq \tau_{\phi }.$$

The tolerance $\tau_{\phi }$ is functional-dependent because heterogeneous characteristics have different scales, physical meanings, and admissible fluctuation ranges. Consequently, the common normalized threshold used later in the experiments serves as an operational reporting point for method comparison across heterogeneous functionals.

The present study focuses on direct feature inference attacks under a one-shot release setting; broader adaptive and learning-based attacks are discussed in Section 4.8. The SCEM interface defined below supports both deterministic and randomized operators, although the representative implementations evaluated in this study are deterministic for reproducibility.

Utility is described by a collection of non-sensitive or task-related functionals $\Psi =\{\psi_{1},\psi_{2},\ldots ,\psi_{M}\}$. These functionals may encode value ranges, non-sensitive spectral amplitudes, selected statistical moments, local ordering, or task-related temporal descriptors. Given functional-specific discrepancy measures $d_{j}$ and tolerances $\delta ={\left[\delta_{1},\ldots ,\delta_{M}\right]}^{⊤}$, the utility-feasible set is

(4) $$\Omega_{u}\left(X;\Psi ,\delta \right)=\left\{Z\in X\;|\;d_{j}\left(\psi_{j}\left(Z\right),\psi_{j}\left(X\right)\right)\leq \delta_{j},\;j=1,\ldots ,M\right\}.$$

Let $\vartheta_{\phi }\in Y_{\phi }$ denote a privacy target, such as a non-sensitive spectral level, an alternative statistical value, or an admissible target interval. The corresponding privacy-target set is

(5) $$\Omega_{p}\left(\vartheta_{\phi };\varepsilon_{\phi }\right)=\left\{Z\in X\;|\;d_{\phi }\left(\phi \left(Z\right),\vartheta_{\phi }\right)\leq \varepsilon_{\phi }\right\},$$

where $\varepsilon_{\phi }$ specifies the desired proximity to the privacy target.

Feature-selective protection is feasible only when the privacy and utility requirements are compatible:

(6) $$\Omega_{p}\left(\vartheta_{\phi };\varepsilon_{\phi }\right)\cap \Omega_{u}\left(X;\Psi ,\delta \right)\neq ⌀.$$

Equation (6) makes the privacy–utility boundary explicit. If the same functional is simultaneously a sensitive target and an indispensable forecasting feature, arbitrarily strong obfuscation and zero utility loss cannot generally be achieved at the same time. In such cases, the admissible protection strength must be selected according to the application-specific utility tolerance.

The problem considered in this paper is therefore to construct a feature-conditioned operator

(7) $$X^{†}=F_{\phi }\left(X;\Theta_{\phi }\right),$$

where $\Theta_{\phi }$ collects the representation, perturbation, recovery, calibration, and privacy strength parameters. The operator should move the selected sensitive functional toward its privacy target while returning a valid sequence inside the utility-feasible set.

For multivariate sensor data $X\in R^{T\times D}$, the current implementation applies SCEM channel-wise. Each channel may correspond to a sensor variable, sensing modality, or monitored physical quantity. Joint multivariate protection can be constructed within the same formulation by defining ϕ over several channels—for example, through cross-variable correlations, shared periodic patterns, or multichannel operational indicators.

### 3.2. Generalized Privacy Energy and Overall Design

SCEM provides a common operator interface through which heterogeneous sensitive functionals can be represented, perturbed, recovered, and calibrated. The framework does not require every functional to share an identical closed-form perturbation equation. Instead, each functional-specific instantiation must provide a compatible set of operators with clearly defined responsibilities.

For a selected functional ϕ, let $Z_{\phi }=M_{\phi }\left(X\right)\in Z_{\phi }$ denote a representation in which the target functional becomes explicit or controllable. Depending on ϕ, $Z_{\phi }$ may be an amplitude spectrum, a centered dispersion sequence, a normalized signal, a local order statistic neighborhood, or a standardized distributional state.

Let $r_{\phi }\in Z_{\phi }$ denote a privacy reference in the representation space. SCEM defines the generalized privacy energy as

(8) $$E_{\phi }=E_{\phi }\left(X;\omega_{\phi },r_{\phi }\right)=\omega_{\phi }⊙{\left[D_{\phi }\left(M_{\phi }\left(X\right),r_{\phi }\right)\right]}^{⊙2},$$

where $\omega_{\phi }\geq 0$ is a structured weight, $D_{\phi }$ is a representation-dependent discrepancy operator, ⊙ denotes the Hadamard product, and ^⊙2^ denotes component-wise squaring. By construction, the generalized privacy energy is component-wise non-negative whenever $\omega_{\phi }\geq 0$, because the discrepancy is squared component-wise before multiplication by the non-negative structured weight. The term “energy” in SCEM therefore denotes a non-negative discrepancy representation relative to a privacy reference. It is not restricted to a single physical unit. Spectral power, centered dispersion energy, and squared functional deviation are specific realizations of Equation (8).

A functional-specific SCEM instantiation is represented by the tuple

(9) $$I_{\phi }=\left(\phi ,M_{\phi },r_{\phi },E_{\phi },T_{\phi },B_{\phi },C_{\phi },\Theta_{\phi }\right),$$

where $T_{\phi }$ is the structured perturbation operator, $B_{\phi }$ is the recovery or back-projection operator, and $C_{\phi }$ is the utility-oriented calibration operator.

The complete workflow is written as

(10) $$\begin{array}{cc} Z_{\phi }\; & =M_{\phi }\left(X\right),\\ E_{\phi }\; & =E_{\phi }\left(X;\omega_{\phi },r_{\phi }\right),\\ {\tilde{Z}}_{\phi }\; & =T_{\phi }\left(Z_{\phi },E_{\phi };\theta_{\phi },\xi_{\phi }\right),\\ \tilde{X}\; & =B_{\phi }\left({\tilde{Z}}_{\phi };X\right),\\ X^{†}\; & =C_{\phi }\left(\tilde{X};X,\Omega_{u}\right). \end{array}$$

Here, $\theta_{\phi }$ controls the perturbation strength or target behavior, and $\xi_{\phi }$ is an optional random variable. A fixed $\xi_{\phi }$ gives a deterministic instantiation, whereas a sampled $\xi_{\phi }$ permits randomized references, weights, perturbation directions, or calibration choices without changing the common operator interface.

**Figure 1:**
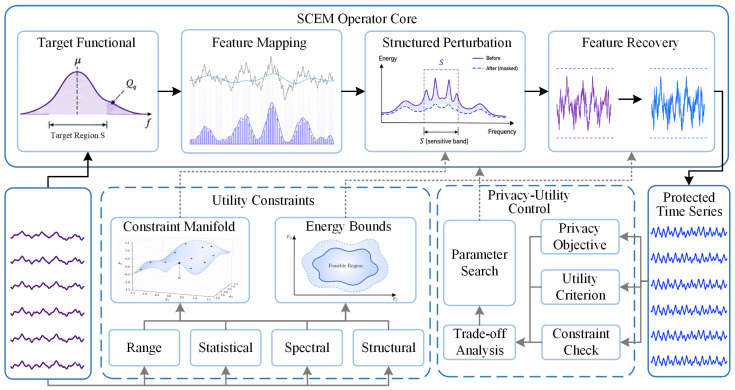
Overall architecture of the SCEM framework. The core workflow consists of target functional specification, feature mapping, structured perturbation, feature recovery, and utility-oriented calibration. The generalized privacy energy quantity is an intermediate discrepancy representation supporting structured perturbation, and utility constraints correspond to the utility-feasible set and calibration stage.

As shown in Figure 1, the four operator stages have different responsibilities. The mapping stage determines where the sensitive functional can be represented. The perturbation stage determines how the privacy-related representation should be modified, using the generalized privacy energy quantity where appropriate as an intermediate discrepancy representation rather than as an additional workflow stage. The recovery stage reconstructs a valid time-series sequence from the perturbed representation. The calibration stage enforces range, statistical, spectral, structural, or task-related utility constraints. This separation is the basis of SCEM’s generality: the operator interface remains invariant, whereas the internal realization of each operator is allowed to depend on the protected functional.

**Definition 1** (Admissible SCEM Instantiation)**.** *A tuple $I_{\phi }$ in Equation (9) is an admissible SCEM instantiation if it satisfies the following conditions:*

*1.*  *Functional representability. There exists a map $g_{\phi }$ such that $\phi \left(X\right)=g_{\phi }\;\left(M_{\phi }\left(X\right)\right)$.*
*2.*  *Non-negativity and reference consistency. The generalized privacy energy is component-wise non-negative, $E_{\phi }\left(X;\omega_{\phi },r_{\phi }\right)\geq 0$, and zero discrepancy corresponds to the selected reference state on the weighted support.*
*3.*  *Bounded and controllable perturbation. For admissible parameters, $T_{\phi }$ maps bounded representations to bounded representations and varies continuously with the admissible control parameter. A quantitative parameter stability condition is stated in the subsequent theorem.*
*4.*  *Recovery of a valid sequence. The recovery operator satisfies $B_{\phi }\left({\tilde{Z}}_{\phi };X\right)\in X$. It need not be the exact mathematical inverse of $M_{\phi }$, but it must return a valid sequence consistent with the selected instantiation.*
*5.*  *Utility-feasible calibration. The calibrated output satisfies*
(11) $$C_{\phi }\left(\tilde{X};X,\Omega_{u}\right)\in \Omega_{u}\left(X;\Psi ,\delta \right).$$
*6.*  *Privacy–utility compatibility. The privacy target and utility constraints satisfy Equation (6). Otherwise, the privacy target or utility tolerances must be relaxed.*

These conditions clarify the meaning of framework generality: SCEM obtains its generality from a shared admissible operator interface, while the representation, privacy reference, perturbation rule, recovery operator, and calibration constraints remain customizable for each functional. The calibration condition directly ensures that the final protected sequence belongs to the prescribed utility-feasible set.

For a vector-valued sensitive functional $\phi ={\left[\phi_{1},\ldots ,\phi_{K}\right]}^{⊤}$, a joint instantiation can be constructed by block-wise concatenation:

(12) $$\begin{array}{cc} M_{\phi }\left(X\right)\; & =\mathrm{concat}\left(M_{\phi_{1}}\left(X\right),\ldots ,M_{\phi_{K}}\left(X\right)\right),\\ E_{\phi }\; & =\mathrm{concat}\left(\lambda_{1}E_{\phi_{1}},\ldots ,\lambda_{K}E_{\phi_{K}}\right),\;\lambda_{k}\geq 0. \end{array}$$

This construction permits combined protection objectives without forcing heterogeneous functionals to have the same representation dimension. The perturbation and calibration operators must then satisfy the corresponding joint privacy and utility constraints.

### 3.3. Representative Instantiations and Extensibility of SCEM

This subsection presents five representative instantiations spanning spectral, first-order statistical, second-order statistical, order-statistical, and high-order distributional functionals. Although their internal operators are functional-specific, all five cases follow the same mapping–perturbation–recovery–calibration interface supported by a generalized privacy energy representation.

For sensitive frequency amplitudes associated with periodic behavior or recurrent operating patterns, SCEM operates in the spectral domain. The original sequence is transformed as

(13) $$\hat{X}=F\left(X\right),\;A=\left|\hat{X}\right|,\;P=∠\hat{X},$$

where *A* and *P* denote the amplitude and phase spectra, respectively. Let *S* denote the sensitive frequency set, and let $A_{\mathrm{bg},k}$ denote the non-sensitive background amplitude estimated from neighboring frequencies outside *S*. The frequency privacy energy is

(14) $$E_{f,k}=w_{f,k}{\left(A_{k}-A_{\mathrm{bg},k}\right)}^{2},\;k\in S,$$

where $w_{f,k}$ emphasizes the sensitive region.

A reference-oriented contraction is applied to the sensitive amplitudes:

(15) $${\tilde{A}}_{k}=A_{\mathrm{bg},k}+\alpha_{f}\left(A_{k}-A_{\mathrm{bg},k}\right),\;k\in S,$$

where $\alpha_{f}\in \left[0,1\right]$. Smaller values of $\alpha_{f}$ move the sensitive amplitudes closer to the background level. Frequencies outside *S* are left unchanged except for optional boundary smoothing.

The preliminary protected sequence is reconstructed by

(16) $$\tilde{X}=F^{-1}\left(\tilde{A}⊙e^{iP}\right).$$

Range correction and non-sensitive spectral calibration are then applied to limit unintended distortion outside the protected frequency region.

For mean-related functionals, the protected mean level may reveal operating states, load levels, or long-term environmental conditions. Let $u_{t}\in \left[0,1\right]$ denote the normalized sample value, let $\mu_{u}$ denote its mean, and let $\mu_{\mathrm{ref}}$ denote the target mean. Define $\Delta_{\mu }=\mu_{\mathrm{ref}}-\mu_{u}$. A bounded adjustment weight $w_{\mu }\left(t\right)\geq 0$ is selected such that

(17) $$\frac{1}{T}\sum_{t=1}^{T}w_{\mu }\left(t\right)=1.$$

The preliminary protected value is

(18) $${\tilde{u}}_{t}=u_{t}+\Delta_{\mu }w_{\mu }\left(t\right).$$

Before range projection, Equation (17) yields $\mu \left(\tilde{u}\right)=\mu_{u}+\Delta_{\mu }$. The weight design assigns smaller changes to samples near the admissible boundaries and larger changes to samples with greater adjustment capacity. The sequence is subsequently mapped back to the original scale and calibrated with respect to value range and selected non-sensitive moments. Rank consistency can be imposed as an optional constraint when ordering is utility-relevant.

For variance-related functionals, protection is formulated in the centered dispersion energy space. Let $z_{t}=x_{t}-\mu$ and $e_{t}=z_{t}^{2}$, where μ is the sample mean. Let $\sigma_{\mathrm{ref}}^{2}$ denote the target variance, and let $q_{v}\left(t\right)\geq 0$ satisfy $\sum_{t=1}^{T}q_{v}\left(t\right)=1$. The required total dispersion change is $\Delta E_{v}=T\left(\sigma_{\mathrm{ref}}^{2}-\sigma^{2}\right)$. The redistributed dispersion energy is

(19) $${\tilde{e}}_{t}=\max\left(e_{t}+\Delta E_{v}q_{v}\left(t\right),\epsilon \right).$$

The preliminary protected sequence is reconstructed by preserving the original centered sign:

(20) $${\tilde{x}}_{t}=\mu +\mathrm{sign}\left(z_{t}\right)\sqrt{{\tilde{e}}_{t}}.$$

Mean correction and variance rescaling are applied after reconstruction. When the lower truncation in Equation (19) is inactive, the redistributed total energy matches the specified target exactly; otherwise, the calibration stage restores the intended variance within the prescribed tolerance.

For quantile and median functionals, the protected values are determined by local order statistics and therefore require a representation concentrated around the selected order-statistic region. Let $Q_{q}\left(u\right)$ denote the *q*-quantile of a normalized sequence *u*, and let $Q_{q,\mathrm{ref}}$ denote the target quantile. A bounded localization kernel $\kappa_{q}\left(\cdot ;s_{q}\right)$ is used to define

(21) $${\bar{w}}_{q}\left(t\right)=\kappa_{q}\left(\left|u_{t}-Q_{q}\left(u\right)\right|;s_{q}\right),\;w_{q}\left(t\right)=\frac{{\bar{w}}_{q}\left(t\right)}{\max_{j}{\bar{w}}_{q}\left(j\right)+\epsilon },$$

where $s_{q}>0$ controls the width of the affected neighborhood. The adjustment direction is $d_{q}=\mathrm{sign}\left(Q_{q,\mathrm{ref}}-Q_{q}\left(u\right)\right)$, and the preliminary local adjustment is

(22) $${\tilde{u}}_{t}=u_{t}+\eta_{q}d_{q}w_{q}\left(t\right),$$

where $\eta_{q}\geq 0$ controls the adjustment magnitude. Because the weight is concentrated near $Q_{q}\left(u\right)$, the transformation modifies the selected order-statistic region while limiting changes to distant samples. The recovered sequence is projected onto the admissible range and, when required, reassigned through an order-consistent correction. The median is obtained as the special case q=0.5.

For high-order moment functionals, skewness and kurtosis characterize distributional asymmetry and tail behavior. SCEM first standardizes the sequence as z=(X−μ1)/(σ+ϵ), where μ and σ are the sample mean and standard deviation. A sign-preserving moment-shaping transformation is then applied:

(23) $$z^{†}=\mathrm{sign}\left(z\right)⊙{\left(|z|+\epsilon \right)}^{\gamma_{m}},$$

where $\gamma_{m}>0$ controls the deformation of central and tail samples, and $\gamma_{m}=1$ corresponds approximately to the identity transformation. In the evaluated implementation, $\gamma_{m}$ is generated from the user-level perturbation strength coefficient $p_{m}$ through a fixed moment-shaping rule; Section 4.1 therefore reports the user-controlled coefficient $p_{m}$. The preliminary reconstruction is

(24) $$\tilde{X}=\mu 1+\sigma z^{†}.$$

The calibration stage corrects the mean, variance, and admissible range so that the high-order distributional shape is modified without introducing unnecessary first- or second-order drift.

These five cases span spectral, first-order, second-order, order-statistical, and high-order distributional functionals under the same admissible operator interface. Additional functionals can be incorporated by specifying the corresponding representation, reference, perturbation, recovery, and calibration operators.

### 3.4. Framework Properties and Theoretical Boundaries

This subsection states the properties that follow from the common SCEM interface and distinguishes them from properties that require additional instantiation-specific assumptions.

Let $E_{\phi ,\mathrm{ref}}$ denote the selected reference state in the generalized privacy energy space; when Equation (8) measures discrepancy directly from $r_{\phi }$, the natural reference is $E_{\phi ,\mathrm{ref}}=0$.

**Proposition 1** (Reference-Oriented Contraction)**.** *For reference-oriented affine energy perturbation, consider the operator*

(25) $$T_{\phi }^{E}\left(E;\beta_{\phi }\right)=\beta_{\phi }E+\left(1-\beta_{\phi }\right)E_{\phi ,\mathrm{ref}},\;\beta_{\phi }\in \left[0,1\right].$$

*Then,*

(26) $$∥T_{\phi }^{E}\left(E;\beta_{\phi }\right)-E_{\phi ,\mathrm{ref}}∥=\beta_{\phi }∥E-E_{\phi ,\mathrm{ref}}∥.$$

**Proof.**  Subtracting $E_{\phi ,\mathrm{ref}}$ from both sides of Equation (25) gives $T_{\phi }^{E}\left(E;\beta_{\phi }\right)-E_{\phi ,\mathrm{ref}}=\beta_{\phi }\left(E-E_{\phi ,\mathrm{ref}}\right)$. Taking the norm yields Equation (26). □

**Corollary 1** (Spectral Energy Contraction)**.** *Using Equations (14) and (15), the perturbed spectral privacy energy satisfies*

(27) $${\tilde{E}}_{f,k}=w_{f,k}{\left({\tilde{A}}_{k}-A_{\mathrm{bg},k}\right)}^{2}=\alpha_{f}^{2}E_{f,k}.$$

*Therefore, $\alpha_{f}$ is the amplitude-space contraction factor, $\alpha_{f}^{2}$ is the corresponding energy-space contraction factor, and $\beta_{f}=\alpha_{f}^{2}$ in the notation of the preceding proposition.*

**Theorem 1** (Boundedness and Parameter Stability)**.** *Assume that the input domain and admissible parameter set are bounded and that $M_{\phi }$, $E_{\phi }$, $T_{\phi }$, $B_{\phi }$, and $C_{\phi }$ are bounded on their respective admissible domains. Then, $F_{\phi }$ maps bounded inputs to bounded outputs. Assume further that the operators $M_{\phi }$, $E_{\phi }$, $T_{\phi }$, $B_{\phi }$, and $C_{\phi }$ are jointly Lipschitz-continuous with respect to their inputs and the admissible parameter $\theta_{\phi }$, uniformly over the bounded admissible domains, with all other admissible parameters held fixed. Then, there exists a finite constant $L_{\phi }$ such that*

(28) $$∥F_{\phi }\left(X;\theta_{1}\right)-F_{\phi }\left(X;\theta_{2}\right)∥\leq L_{\phi }\left|\theta_{1}-\theta_{2}\right|.$$

**Proof.**  Boundedness follows from the composition of bounded maps. The Lipschitz result follows by repeatedly applying the Lipschitz inequality through the complete operator workflow, including the privacy energy construction. The constant $L_{\phi }$ is obtained by recursively combining the input-Lipschitz and parameter-Lipschitz constants of the component operators over the bounded admissible domains. □

**Proposition 2** (Perturbation–Calibration Displacement Bound)**.** *Assume that $d_{\phi }$ is a metric or a norm-induced discrepancy. Let $\tilde{X}$ denote the recovered sequence before calibration, and define*

(29) $$\begin{array}{cc} m_{\phi }\; & =d_{\phi }\left(\phi \left(\tilde{X}\right),\phi \left(X\right)\right),\\ \kappa_{\phi }\; & =d_{\phi }\left(\phi \left(X^{†}\right),\phi \left(\tilde{X}\right)\right). \end{array}$$

*where $m_{\phi }$ is the pre-calibration functional displacement and $\kappa_{\phi }$ is the additional displacement introduced by calibration. Then,*

(30) $$d_{\phi }\left(\phi \left(X^{†}\right),\phi \left(X\right)\right)\geq m_{\phi }-\kappa_{\phi }.$$

**Proof.**  The result follows from the reverse triangle inequality applied to $d_{\phi }$. □

Equation (30) clarifies the roles of the SCEM stages: mapping identifies the controllable representation, structured perturbation and recovery jointly create the pre-calibration privacy-related displacement, and calibration limits utility distortion.

**Remark 1** (Instantiation-Specific Structural Constraints)**.** *Additional structural properties can be incorporated as instantiation-specific utility constraints. For example, when ordering is utility-relevant, the utility-feasible set may additionally require argsort(Z)=argsort(X).*

**Remark 2** (Deterministic and Randomized Instantiations)**.** *The optional variable $\xi_{\phi }$ in Equation (10) allows the randomization of the privacy reference, structured weights, perturbation direction, or calibration choices. The deterministic instantiations used in the present experiments facilitate reproducibility and controllability, while randomized variants can be designed for repeated-observation or mapping dictionary threat models.*

Together, the preceding results establish non-negative generalized privacy energy, reference-oriented contraction, output feasibility, bounded parameter behavior, and a stage-wise displacement bound for admissible SCEM instantiations.

### 3.5. Privacy–Utility Behavior and Complexity Analysis

SCEM treats privacy protection and utility preservation as coupled objectives. Stronger perturbation generally moves the selected functional farther from its original value or closer to its privacy reference but may increase the distortion of utility-related structures. The admissible protection strength is therefore determined by the compatibility condition in Equation (6), rather than by a universal parameter setting shared across all instantiations.

For mean shifting, variance redistribution, and moment shaping, feature construction, perturbation, recovery, and one calibration pass are linear in the sequence length. With *C* calibration iterations, the complexity is O(T)+O(CT).

For spectral instantiations, the dominant operations are the Fourier transform and inverse reconstruction, giving a complexity of O(TlogT)+O(CT).

For quantile-related instantiations, full sorting has complexity O(TlogT), whereas selection-based or pre-sorted local implementations can reduce the order-statistic search cost. Optional projection onto a small number of task-related bases adds a cost proportional to the number of retained bases.

For a *D*-channel dataset processed independently, the corresponding channel-wise cost scales linearly with *D*. SCEM does not require the training of a generative or predictive model as part of the protection mechanism. Its operator-based complexity suggests potential suitability for large-scale preprocessing and resource-constrained deployment, although device-level latency and energy consumption require platform-specific validation.

In summary, SCEM separates privacy targets, utility constraints, and functional-specific operators within a shared admissible interface, providing a basis for extensible structure-preserving time-series protection.

## 4. Experimental Design and Result Analysis

### 4.1. Experimental Setup

To evaluate SCEM, we use two sensor-related monitoring benchmarks, ETTm1 and Weather, and one non-sensor temporal benchmark, Exchange Rate, for cross-domain evaluation [44,45]. ETTm1 contains 7 variables collected at 15 min intervals with 69,680 time steps; Weather contains 21 meteorological variables recorded at 10 min intervals with 52,696 time steps; and Exchange Rate contains 8 daily exchange rate variables with 7588 time steps. ETTm1 and Weather represent energy or industrial monitoring and environmental sensing scenarios, respectively, whereas Exchange Rate is included to examine whether the operator interface design remains applicable outside physical sensing scenarios under the same evaluated protocol.

SCEM is evaluated through five representative instantiations covering sensitive frequency amplitudes, mean, variance, median, and high-order moments. The reported experiments instantiate one sensitive functional at a time so that the behavior of each protection mechanism can be examined separately. For multivariate datasets, SCEM is applied channel-wise to a fixed set of target variables selected before evaluation. The same variables, input sequences, attack windows, and downstream forecasting protocol are used for all protection methods. Statistical feature inference attacks were evaluated over normalized windows. For ETTm1 and Weather, the analysis used the first 20 non-overlapping windows, each containing 2000 samples; for Exchange Rate, the analysis used 20 sliding windows of length 2000 with stride of 200.

Three representative baselines covering random perturbation, statistic-level differential privacy, and time-series anonymization are considered. Additive Gaussian Noise (AGN) is implemented as

(31) $$X_{\mathrm{AGN}}=X+\eta ,\;\eta_{t}\overset{i.i.d.}{∼}N\left(b,\sigma^{2}\right),\;b=x_{\max}-x_{\min},\;\sigma =0.1b,$$

with random seed 42. Thus, *b* is the Gaussian mean bias set to the data range, rather than a clipping bound. The statistic-level sliding-window Laplace baseline perturbs window means with nominal privacy budget $\epsilon_{\mathrm{DP}}=0.5$, window length 48, and scale $\Delta f/\epsilon_{\mathrm{DP}}$, where $\Delta f=(x_{\max}-x_{\min})/48$. Because overlapping windows repeatedly query shared samples, this method is treated as a heuristic statistic-level DP reference rather than a strict sequence-level DP mechanism. The k-nTS baseline uses segmentation and clustering-based anonymization with k=3, window length m=20, and step size 10 [32].

Min–max normalization is applied before downstream model training. For local statistical feature inference evaluation, normalization is performed independently within each attack window, as defined in Equation (33). The sensitivity experiment varies the reference-oriented control parameter over α∈{0.1,0.2,…,0.9}. All reported parameters are fixed before evaluation and are not tuned separately for individual evaluation windows.

For the representative ETTm1 spectral instantiation, the sensitive frequency is set to 2 cycles/day with a sensitive-band tolerance of ±0.03 cycles/day. The background spectrum is estimated from the neighboring region within ±0.1 cycles/day, excluding the sensitive band. Sensitive amplitudes are adjusted using the background mean and standard deviation with a structural scaling coefficient of 0.5, followed by Gaussian smoothing with σ=0.3. The protected sequence is reconstructed by inverse FFT while retaining the original phase and is subsequently calibrated to preserve the original mean.

For the representative statistical SCEM instantiations, the mean-related configuration used the proportional adjustment mode. The shift coefficients were 0.75 for ETTm1, 1.0 for Weather, and 0.5 for Exchange Rate, corresponding to target means $\mu_{\mathrm{ref}}=1.75\mu$, $\mu_{\mathrm{ref}}=2.0\mu$, and $\mu_{\mathrm{ref}}=1.5\mu$, respectively. The variance-related configuration used $\gamma_{v}=0.5$ for ETTm1 and $\gamma_{v}=1.0$ for Weather and Exchange Rate, yielding target variances $\sigma_{\mathrm{ref}}^{2}=1.5\sigma^{2}$ and $\sigma_{\mathrm{ref}}^{2}=2.0\sigma^{2}$, respectively. For median protection, the target quantile was fixed at q=0.5, with a localization width parameter of $s_{q}=0.5$, a privacy energy contraction coefficient of 0.3, and a back-projection step ratio of 0.5 for all three datasets; the orthogonal perturbation amplitude was 200 for ETTm1 and Weather and 20 for Exchange Rate. The high-order moment instantiation used a unified perturbation strength coefficient of $p_{m}=0.1$ for the skewness- and kurtosis-related transformations. These settings were fixed before evaluation and were not tuned separately for individual evaluation windows.

The downstream utility evaluator is a one-layer LSTM implemented in PyTorch. For each prediction sample, the input length is set to 288 time steps, and the output is a 24-step-ahead prediction vector. The input dimension equals the number of variables used by the corresponding dataset, and the hidden dimension of the LSTM is set to 50. The last hidden output of the LSTM is passed to a fully connected linear layer to generate the 24-dimensional prediction output. The batch size is 32, and the model is trained for 50 epochs. Adam is used as the optimizer, with a learning rate of 0.001, and the mean squared error is used as the training loss. The samples are divided chronologically into 80% training data and 20% validation data after min–max normalization to [0,1]. The training mini-batches are shuffled, whereas the validation mini-batches are not shuffled. No dropout, early stopping, learning rate scheduler, or dataset-specific architecture adjustment is used in the reported experiments. The same architecture, data split, and training protocol are used for the original and all protected datasets.

Feature-level privacy is evaluated using the attack success rate (ASR), relative difference rate (RDR), and peak ratio threshold (PRT). Forecasting-oriented utility is assessed using the $R^{2}$, MAE, RMSE, and MAPE (%). All experiments are conducted on a workstation equipped with an Intel Core i5-14600KF CPU and an NVIDIA RTX 5070 GPU with 12 GB memory. The implementation uses Python 3.9, NumPy 1.24, SciPy 1.10, Scikit-learn 1.2, and Matplotlib 3.7.

### 4.2. Evaluation Metrics

We evaluate protection from two complementary perspectives: the recoverability of predefined sensitive functionals and the preservation of forecasting-oriented utility.

The primary privacy metric is the attack success rate:

(32) $$\mathrm{ASR}=\frac{N_{\mathrm{success}}}{N_{\mathrm{total}}}\times 100\%,$$

where $N_{\mathrm{success}}$ and $N_{\mathrm{total}}$ denote the numbers of successful inference trials and total evaluated trials, respectively. A lower ASR indicates lower recoverability of the selected functional under the evaluated attack protocol.

For statistical feature inference, all features are computed after window-wise min–max normalization:

(33) $$x_{\mathrm{norm}}=\frac{x-\min(X_{w})}{\max\left(X_{w}\right)-\min\left(X_{w}\right)+\epsilon },$$

where $X_{w}$ is the local evaluation window and ϵ is a small numerical constant. This normalization reduces the influence of trivial scale differences and yields a more demanding feature inference setting focused on a normalized statistical structure.

For a statistical functional ϕ, the normalized relative discrepancy is

(34) $$\Delta_{\phi }\left(X_{p},X_{o}\right)=\frac{\left|\phi \left(X_{p}^{\mathrm{norm}}\right)-\phi \left(X_{o}^{\mathrm{norm}}\right)\right|}{\left|\phi (X_{o}^{\mathrm{norm}})\right|+\epsilon }\times 100\%,\;\phi \in \left\{\mu ,\sigma^{2},Q_{0.5},\mathrm{Skew},\mathrm{Kurt}\right\}.$$

A statistical inference trial is counted as successful when

(35) $$\Delta_{\phi }\left(X_{p},X_{o}\right)\leq \tau_{\phi }.$$

Because $\Delta_{\phi }$ is expressed as a relative percentage discrepancy, the choice $\tau_{\phi }=10\%$ provides a dimensionless common numerical operating point for comparative reporting across the evaluated statistical functionals. It is not interpreted as a universal physical tolerance. In an application-specific deployment, a functional-dependent attack tolerance vector $\tau ={\left[\tau_{\phi_{1}},\ldots ,\tau_{\phi_{K}}\right]}^{⊤}$ should be selected according to sensor precision, natural variability, domain risk, and the operational meaning of each protected functional. The resulting metric is denoted as ASR@10%.

For frequency-domain inference, let $A_{\mathrm{target}}$ be the amplitude of the target sensitive frequency and $A_{\max}$ the maximum spectral amplitude. The peak ratio is

(36) $$\mathrm{PRT}=\frac{A_{\mathrm{target}}}{A_{\max}}\times 100\%.$$

A frequency-domain inference trial is counted as successful when $\mathrm{PRT}\geq \tau_{f}$. The same numerical value, $\tau_{f}=10\%$, is used as the reporting point for frequency-domain inference. However, $\tau_{f}$ measures spectral prominence through PRT rather than the relative statistical reconstruction error. Therefore, the statistical and frequency-domain criteria share a common numerical operating point but not a common physical interpretation.

The relative difference rate is ${\mathrm{RDR}}_{\phi }=\Delta_{\phi }\left(X_{p},X_{o}\right)$. For a privacy-sensitive functional, a larger RDR indicates stronger modification of the original functional. For a utility-related non-sensitive functional, a smaller RDR indicates better preservation. RDR is therefore interpreted together with the ASR and downstream forecasting results rather than as a standalone measure.

Forecasting-oriented utility is measured using a fixed LSTM evaluator [46]; a higher $R^{2}$ and lower MAE, RMSE, and MAPE (%) indicate better predictive utility under the adopted protocol.

### 4.3. Forecasting-Oriented Utility Analysis

The utility analysis examines temporal trajectories, feature distributions, low-dimensional visualizations, feature-level shifts, and downstream forecasting results.

**Figure 2:**
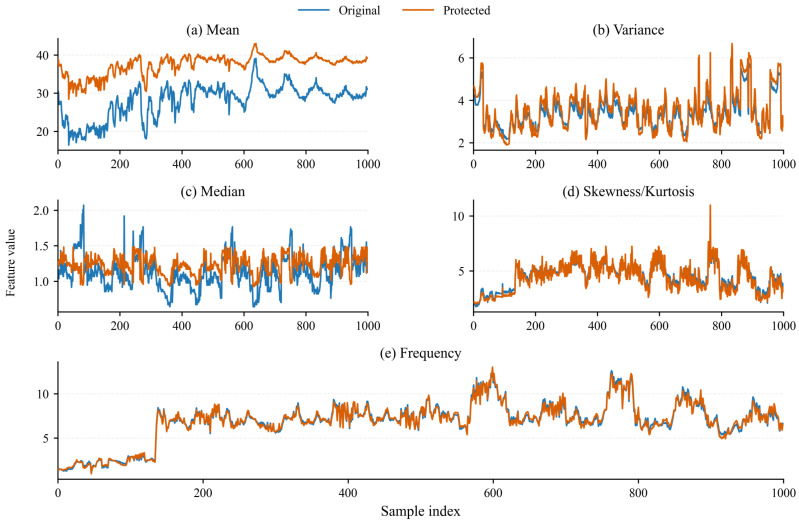
Temporal comparison of original and protected features over the evaluated segment.

Figure 2 shows that SCEM modifies the designated sensitive characteristics while retaining the overall temporal evolution of the evaluated sequences.

**Figure 3:**
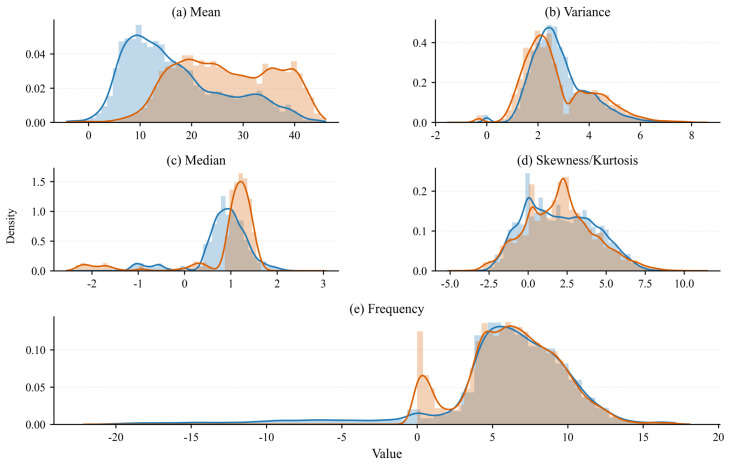
Distributional comparison of original and protected features.

Figure 3 shows visible shifts in selected sensitive distributions together with substantial overlap in several utility-related characteristics.

**Figure 4:**
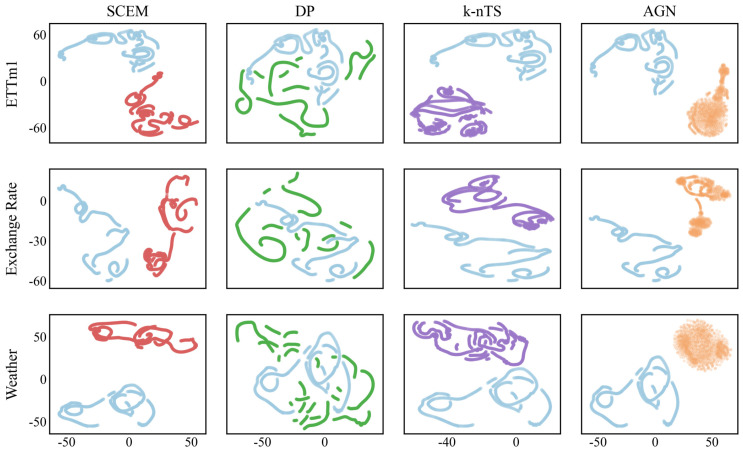
t-SNE visualization of original and protected time-series representations. The visualization provides a qualitative comparison of the low-dimensional organization of original and protected time-series representations.

Figure 4 provides qualitative evidence that SCEM retains a low-dimensional organization that is closer to the original data than the evaluated baselines.

**Figure 5:**
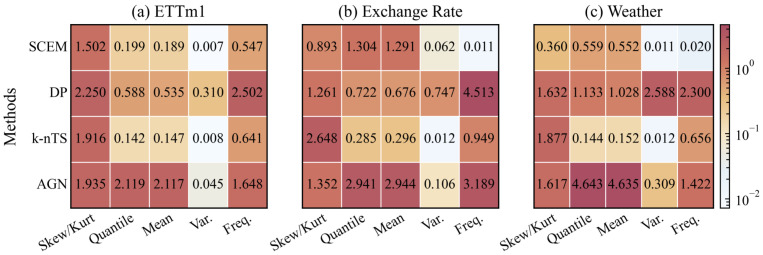
Relative feature shift ratio across datasets and protection methods, calculated using Equation (37). The numerical annotations are original untransformed relative ratios; for example, 1.0 corresponds to a 100% relative shift. Heatmap colors use logarithmic normalization to improve visual discrimination across different magnitudes.

For Figure 5, the displayed feature shift value is defined as the unscaled relative ratio

(37) $$S_{\phi }\left(X_{p},X_{o}\right)=\frac{\left|\phi \left(X_{p}\right)-\phi \left(X_{o}\right)\right|}{\left|\phi (X_{o})\right|+\epsilon }.$$

Thus, a displayed value of 1.502 corresponds to a relative shift of 150.2%, whereas 0.199 corresponds to 19.9%. The numerical annotations show the original untransformed relative ratios, whereas the heatmap colors are displayed using logarithmic normalization to improve visual discrimination across different magnitudes. The values are comparable within the same functional and dataset, but their absolute magnitudes should not be compared across heterogeneous functionals without considering their different scales and meanings.

Figure 5 shows that SCEM produces non-uniform changes across features rather than applying a single global distortion pattern. Larger shifts appear for designated privacy-sensitive components, whereas several utility-related characteristics remain closer to their original values. This descriptive result is consistent with the feature-conditioned mapping, perturbation, recovery, and calibration design.

Table 1 shows that SCEM retains competitive forecasting-oriented utility across the three datasets. Its performance remains equal or close to that of the original-data reference on Weather and Exchange Rate and is substantially better than DP and AGN on several ETTm1 metrics. Although k-nTS is competitive on selected metrics, the results, together with Table 2, support a favorable privacy–utility balance rather than uniformly optimal forecasting accuracy.

### 4.4. Privacy Attack Experiment

The privacy experiment evaluates whether predefined statistical and spectral functionals remain recoverable from the released sequence under the one-shot feature inference threat model in Section 3.1. Statistical feature inference targets the mean, variance, median, skewness, and kurtosis, while frequency-domain inference tests whether the designated spectral component remains prominent after protection. The primary result is the ASR@10%, with lower values indicating lower recoverability under this protocol.

Table 2 shows that the evaluated SCEM instantiations achieve ASR@10% values no higher than 15% across the 18 reported feature–dataset pairs, with an unweighted mean of 4.69%. Some baselines obtain equal or lower ASR values on individual pairs; therefore, the privacy results should be interpreted jointly with the forecasting utility in Table 1. The reduced recoverability is consistent with the intended roles of spectral contraction, variance redistribution, quantile adjustment, and moment shaping.

### 4.5. Feature-Selective Privacy Sensitivity Analysis

The sensitivity analysis varies the reference-oriented coefficient over α∈[0.1,0.9] to examine gradual feature-level responses. Here, α is a common sensitivity analysis index rather than an identical physical parameter shared by all SCEM instantiations, and smaller values represent stronger movement toward the reference state.

**Figure 6:**
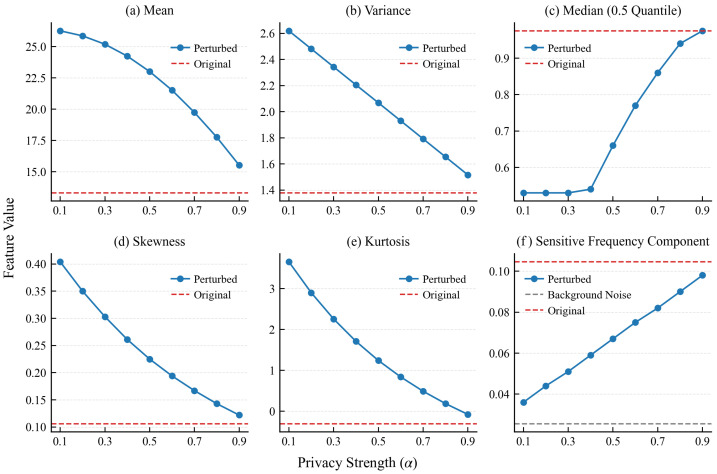
Sensitivity analysis of statistical and frequency characteristics under different values of the reference-oriented privacy control parameter α. The curves show how each protected feature responds to gradually changing privacy strength. The horizontal reference lines indicate the original statistical values and the background noise level in the frequency subplot, serving as comparative baselines rather than hard pass–fail privacy thresholds.

Figure 6 presents an α-sensitivity scan rather than a pass–fail test against fixed privacy target values. The purpose of this analysis is to examine how the protected statistical and spectral characteristics respond when the privacy control parameter varies from 0.1 to 0.9. The results show that different sensitive characteristics exhibit feature-specific response trends: the statistical descriptors change gradually without abrupt oscillations, while the sensitive frequency amplitude varies systematically with respect to the selected background reference.

The sensitivity results therefore support the controllability of SCEM through feature-specific responses to the shared privacy control principle. In this analysis, the reference lines are used only as comparative baselines for interpreting the response trends under different privacy strengths, not as universal thresholds for judging whether protection is successful.

### 4.6. Overall Privacy–Utility Trade-Off

To summarize privacy and utility jointly, the privacy coordinate is calculated as the unweighted mean ASR@10% over the six attacks in Table 2. The utility coordinate is the non-negative relative RMSE increase over the original-data reference:

(38) $$\Delta_{\mathrm{RMSE}}=\max\left(0,\frac{{\mathrm{RMSE}}_{\mathrm{method}}-{\mathrm{RMSE}}_{\mathrm{original}}}{{\mathrm{RMSE}}_{\mathrm{original}}+\epsilon }\right)\times 100\%.$$

The value is clipped at zero because the ordinate measures utility loss, while the equal-weight average provides only an aggregate view alongside the feature-specific results.

**Figure 7:**
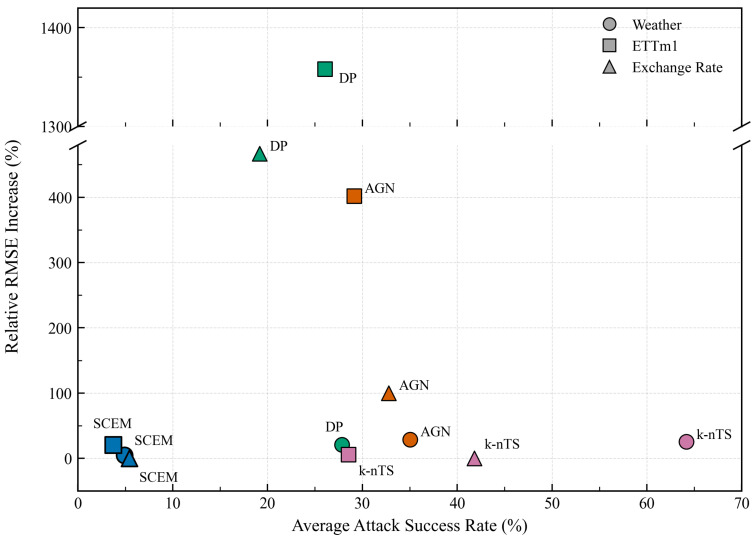
Aggregate privacy–utility trade-off. The x-axis is the unweighted mean ASR@10% over six evaluated attacks, and the y-axis is the non-negative relative RMSE increase over the original-data reference. Lower-left positions indicate a more favorable aggregate trade-off.

Figure 7 places SCEM in a favorable low-leakage and low-to-moderate utility loss region under the adopted aggregate metrics.

### 4.7. Implications for Intelligent Sensor Data Sharing

SCEM can serve as a feature-selective preprocessing layer before time-series data are released to external analysts or cloud-based models. In smart metering, industrial monitoring, environmental sensing, and related IoT settings, its operator interface allows data publishers to modify selected sensitive functionals while constraining utility-related characteristics within prescribed tolerances.

### 4.8. Scope and Limitations

The current evaluation focuses on deterministic, channel-wise SCEM instantiations applied to one predefined functional at a time under direct feature inference attacks. Joint cross-functional and cross-channel protection, randomized instantiations against repeated-observation or surrogate-model attacks, formal individual-level privacy guarantees, and learned attack models require additional mechanism-specific evaluation. SCEM does not provide an individual-level DP guarantee in the reported instantiations, and the statistic-level Laplace baseline is included as a heuristic reference. Device-level latency, energy consumption, and high-dimensional stress testing also remain topics for future deployment studies. Accordingly, the empirical conclusions are limited to the reported datasets, feature-specific instantiations, forecasting evaluator, and attack protocol.

## 5. Conclusions and Future Work

This paper presents SCEM, a unified operator interface framework for feature-selective time-series privacy protection. Its shared mapping–perturbation–recovery–calibration interface supports spectral amplitudes, mean, variance, quantiles, and high-order moments while constraining utility-related structures.

The framework analysis establishes conditional properties including non-negative privacy energy representation, output feasibility, bounded behavior, and parameter stability. Across ETTm1, Weather, and Exchange Rate, SCEM achieved ASR@10% values no higher than 15%, with an unweighted mean of 4.69% over the 18 reported pairs, while maintaining competitive forecasting-oriented utility under the evaluated protocol. Future work will examine joint functionals, randomized instantiations, formal privacy integration, learned attacks, and edge-oriented deployment.

## Figures and Tables

**Table 1:** Forecasting-oriented utility results based on LSTM prediction metrics.

Method	Weather				ETTm1				Exchange Rate			
	$R^{2}$	MAE	RMSE	MAPE (%)	$R^{2}$	MAE	RMSE	MAPE (%)	$R^{2}$	MAE	RMSE	MAPE (%)
Original	0.79	4.95	7.56	1.11	0.90	0.74	1.07	14.30	0.85	0.02	0.03	2.75
SCEM	0.79	5.39	7.93	1.24	0.89	1.28	1.29	4.80	0.85	0.02	0.03	2.85
DP	0.69	5.89	9.14	1.32	0.40	11.90	15.60	95.90	0.02	0.13	0.17	19.10
k-nTS	0.66	6.46	9.47	1.45	0.78	0.72	1.13	5.40	0.71	0.01	0.01	1.49
AGN	0.64	6.72	9.73	1.51	0.23	4.28	5.37	7.50	0.54	0.05	0.06	3.68

**Table 2:** Attack success rate at the common numerical operating point of 10% (ASR@10%) under the evaluated feature inference protocol.

	Weather				ETTm1				Exchange Rate			
	SCEM	DP	k-nTS	AGN	SCEM	DP	k-nTS	AGN	SCEM	DP	k-nTS	AGN
Freq	9.52	57.14	100.00	95.24	7.41	81.48	96.30	100.00	12.50	25.00	95.83	91.67
Mean	5	40	100	65	15	40	20	60	0	65	80	85
Var	0	15	40	5	0	15	20	0	10	0	25	0
Median	15	45	0	45	0	15	20	10	10	20	0	20
Skew	0	0	80	0	0	0	10	5	0	5	40	0
Kurt	0	10	65	0	0	5	5	0	0	0	10	0

## Data Availability

The benchmark datasets used in this study are publicly available. The implementation code for reproducing the main results is publicly available at https://github.com/jshe6427-cpu/SCEM-time-series-privacy (accessed on 12 July 2026).
